# SUPPORTING NURSING HOME STAFF THROUGH PERSON-CENTERED CARE PRACTICES

**DOI:** 10.1093/geroni/igz038.2571

**Published:** 2019-11-08

**Authors:** Diana White, Sarah Dys, Jaclyn Winfree, Serena Hasworth, Ozcan Tunalilar

**Affiliations:** 1 Institute on Aging, Portland, Oregon, United States; 2 Portland State University, Portland, Oregon, United States

## Abstract

Policies and practices have increasingly focused on person-centered care (PCC) to improve quality of life for long-term care residents and staff. Adequate staffing has been a consistent barrier to implementing and sustaining PCC practices. The purpose of this paper is to explore the association between job satisfaction and PCC practices. This research was conducted in a stratified random sample of 33 Oregon nursing homes which were representative in terms of quality, profit/nonprofit ownership, and urban/rural location. Data were collected from 415 staff who completed the staff assessment of person-directed care, direct care worker job satisfaction scale, turnover intention, and organizational belongingness. Consistent with other research, job satisfaction is significantly and negatively correlated with turnover intention (r=-.66) and positively associated with belongingness (r=.66). It is also significantly correlated with scales related to five PCC practices: personhood, autonomy, knowing the person, individualized care, and relationships. Regression analyses examined how these five aspects of PCC practices were associated with 1) job satisfaction and 2) number of deficiencies. Perceptions of practices to support autonomy, personhood, and relationships were associated with higher ratings of job satisfaction among staff. In general, those reporting these practices were in place at least half of the time or with at least half of the residents, showed significantly greater positive associations with job satisfaction (p<.05). Only lower staff reports of autonomy practices were associated with higher deficiencies (p<.05). Findings from this research suggest that supporting PCC practices benefit staff through increased job satisfaction and potentially reduced turnover.

